# Zebrafish-based assessment of luteolin’s potential in modulating seizure responses

**DOI:** 10.3389/fphar.2025.1656301

**Published:** 2025-08-29

**Authors:** Sabrina Ester Schneider, Jefferson Pedroso, Cássia Alves Lima-Rezende, Samara Cristina Mazon, Aline E. dos Santos, Gean Pablo S. Aguiar, Marcelo Lanza, Mariana Appel Hort, J. Vladimir Oliveira, Angelo Piato, Liz Girardi Müller, Anna Maria Siebel

**Affiliations:** ^1^ Curso de Ciências Biológicas, Universidade Comunitária da Região de Chapecó, Chapecó, Brazil; ^2^ Curso de Biomedicina, Universidade Comunitária da Região de Chapecó, Chapecó, Brazil; ^3^ Programa de Pós-Graduação em Ciências Ambientais, Universidade Comunitária da Região de Chapecó, Chapecó, Brazil; ^4^ Departamento de Engenharia Química e de Alimentos, Universidade Federal de Santa Catarina, Florianópolis, Brazil; ^5^ Programa de Pós-Graduação em Ciências da Saúde, Faculdade de Medicina, Universidade Federal do Rio Grande, Rio Grande, Brazil; ^6^ Instituto de Ciências Biológicas, Universidade Federal do Rio Grande, Rio Grande, Brazil; ^7^ Departamento de Farmacologia, Instituto de Ciências Básicas da Saúde, Universidade Federal do Rio Grande do Sul, Porto Alegre, Brazil; ^8^ Instituto de Biologia Experimental e Tecnológica - iBET, Lisboa, Portugal; ^9^ Programa de Pós-Graduação em Farmacologia, Departamento de Farmacologia, Setor de Ciências Biológicas, Universidade Federal do Paraná, Curitiba, Brazil

**Keywords:** epilepsy, luteolin, micronization, seizure, zebrafish

## Abstract

**Introduction:**

Epilepsy is a chronic neurological disorder marked by recurrent seizures. Neuroinflammation and mammalian target of rapamycin (mTOR) signaling are involved in neuronal hyperexcitability, contributing to the onset and persistence of seizures. Repeated seizures during development may cause cellular, cognitive, and behavioral impairment. About 30% of patients do not respond to available treatments, which emphasizes the need for new therapeutic options. Luteolin, a natural compound known for its anti-inflammatory properties and that modulates mTOR, is a promising candidate for seizure control. This study evaluated the antiseizure potential of luteolin and micronized luteolin in zebrafish (*Danio rerio*) larvae exposed to pentylenetetrazole (PTZ).

**Materials and Methods:**

Five-day-old zebrafish larvae were treated with embryo medium (control), diazepam (positive control), luteolin, or micronized luteolin, followed by PTZ exposure. Seizure frequency and intensity were recorded, along with occurrence and latency to seizure stages. Locomotor and behavioral responses were analyzed 24 h later. Brain tissue was used to assess molecular markers of inflammation (*IL-1*β, *IL-6*, *TNF-*α), mTOR signaling (*p70S6Ka*, *p70S6Kb*), and cell condition (*BDNF*, *caspase-3*).

**Results:**

Both luteolin presentations significantly reduced seizure incidence and severity. No locomotor or behavioral changes were observed 24 h after seizures when comparing PTZ-exposed animals to sham groups. Furthermore, molecular analyses revealed no significant changes in the expression levels of the tested markers 24 h after seizures.

**Discussion:**

These findings provide initial evidence that luteolin, in both raw and micronized forms, has antiseizure properties in developing zebrafish. Further research is needed to uncover the pharmacokinetic profile and mechanisms involved.

## 1 Introduction

Epilepsy is a chronic neurological condition characterized by an enduring predisposition to suffering from seizures that are manifested by signs ranging from sensorial disruptions to disturbed motor movements and loss of consciousness (Fisher et al., 2005). Epilepsy presents the highest rates of incidence and prevalence during childhood (Berg et al., 2013; Symonds et al., 2021; Zuberi et al., 2022). Epileptic seizures are unpredictable in occurrence, severity, and duration. Therefore, epileptic children present higher rates of physical problems (as fractures) and an increased risk of premature death (three times higher than in the general children) (World Health Organization, 2024). The susceptibility to have unpredictable seizures promotes psychological comorbidities, including anxiety and depression, promoting enduring damage in the patients’ quality of life (Devinsky et al., 2018). Also, epilepsy during the neurodevelopment stage is frequently associated with cognitive and behavioral comorbidities since recurrent seizures impair brain development and function (Berg et al., 2008; Holmes, 2016; Symonds et al., 2021). Epilepsies of early childhood are recurrently resistant to the available pharmacological therapy, and more than 30% of patients still suffer seizures, being exposed to all risks above-mentioned (World Health Organization, 2024). Therefore, it is necessary to discover innovative treatments that prevent epileptic seizure. Several polyphenols, like luteolin, exhibit promising effects, reducing the seizure severity in rats and mice (Cheng et al., 2024; Luo et al., 2025). However, luteolin’s pharmacological efficacy is often limited by its poor bioavailability (Mao et al., 2025). Interestingly, micronization has emerged as a strategy to improve solubility and absorption, thereby enhancing the therapeutic potential of such compounds (Aguiar et al., 2018; dos Santos et al., 2022; de Oliveira et al., 2023).

Seizures occur as a result of a central excitatory-inhibitory imbalance that promotes abnormally excessive or synchronous neuronal activity episodes (Fisher et al., 2005). Therefore, GABAergic and glutamatergic neurotransmission are antiseizure targets (Bialer and White, 2010). Studies evidence that seizure activity is not reduced to a central excitatory-inhibitory imbalance. Different studies have been showing that neuroinflammation and the mTOR cascade play central roles in seizures and could represent potential targets for epilepsy treatment (Rho and Boison, 2022; Ravizza et al., 2024; Zhang et al., 2024). Inflammatory cascades are activated during the seizure process and aggravate that (Tang et al., 2022; Cheng et al., 2024). Interestingly, pro-inflammatory cytokines (like IL-1β and TNF-α) are higher in drug-resistant epileptic mice than in non-drug-resistant epileptic mice (Tang et al., 2022). In addition, proteins from the mTOR pathway are upregulated in response to seizures and during epileptogenesis, occurring during neurodevelopment (Limanaqi et al., 2020; Ostendorf and Wong, 2015; Talos et al., 2012). Several cytokines (for example, IL-1β, IL-6, and TNF-α) that are found elevated during seizures upregulate the mTOR signaling pathway (Hodges and Lugo, 2020; Ravizza et al., 2024). Mutually, mTOR regulates neurotrophic factors (as BDNF) related to inflammatory responses occurring during seizures (Hodges and Lugo, 2020; Ravizza et al., 2024). Therefore, neuroinflammation and the mTOR complex are critical pathways that are upregulated in brain cells during pharmaco-resistant epilepsies (Ravizza et al., 2024).

Thus, natural products presenting anti-inflammatory properties and targeting mTOR hyperactivation deserve to be investigated for their antiseizure and neuroprotective potential (Ravizza et al., 2024). Luteolin (3′,4′,5,7-tetrahydroxyflavone) is a flavonoid polyphenolic compound found in various plant species, including herbs, fruits, vegetables, and flowers (Zhu et al., 2024). This flavonoid exhibits a range of pharmacological properties, primarily through the modulation of cytokines levels and mTOR signaling (Zhu et al., 2024). In a model of PTZ-induced seizures in adult male Sprague-Dawley rats, luteolin at doses of 20 mg/kg and 50 mg/kg increased seizure latency and decreased seizure duration (Cheng et al., 2024). Moreover, luteolin at 50 mg/kg attenuated spatial learning and memory impairments, as well as anxiety-like behavior, in PTZ-exposed rats (Cheng et al., 2024). This dose also reduced the levels of pro-inflammatory cytokines (TNF-α, IL-6, and IL-1β) and neuronal damage in this model (Cheng et al., 2024). In C57 mice (8–9 weeks old; both sexes), luteolin administered at 120 mg/kg/day for 14 days significantly alleviated lipopolysaccharide (LPS)-induced cognitive deficits by inhibiting the overproduction of inflammatory cytokines in the hippocampus and cortex (Zhou et al., 2021). In microglial cells stimulated by LPS, luteolin at 25, 100, and 200 μM suppressed IL-1β and TNF-α mRNA expression, while the 200 μM dose also reduced these protein levels (Zhou et al., 2021). In human glioma cell lines (U251MG and U87MG), luteolin at 80 μM inhibited mTOR signaling (Anson et al., 2018). Therefore, luteolin has been increasingly investigated as a promising antiseizure and neuroprotective candidate (Garbinato et al., 2021; Cheng et al., 2024; Luo et al., 2025).

Zebrafish (*Danio rerio*) has been widely used in studies that investigate the neurobiological mechanisms related to epileptic seizures and to screen new antiseizure drugs (Baraban, 2021; Szep et al., 2023). Seizures in zebrafish induce behavioral, molecular, and electrographic changes similar to those observed in rodents (Baraban et al., 2005; Baraban, 2021; Chitolina et al., 2023). PTZ-induced seizures in zebrafish larvae are similar to those in rodent models and detect the same targets for seizure control and neuroprotection (Löscher, 2011; Alachkar et al., 2020; Baraban, 2021).

Therefore, considering that neuroinflammation and mTOR signaling play an central role in epileptic seizures and luteolin presents anti-inflammatory properties and targets mTOR hyperactivation but presents low bioavailability, we propose the investigation of the antiseizure and neuroprotective potential of luteolin and micronized luteolin in a PTZ-induced seizure model in zebrafish larvae with the aim of collaborate for identify new therapeutic targets and screen new drugs for the treatment of epilepsy occurring during the early stages of the life.

## 2 Materials and methods

### 2.1 Materials

Luteolin [(2-(3,4-dihydroxyphenyl)-5,7-dihydroxychromen-4-one), 98%, Kingherbs, China] was used in its raw and micronized forms. Acetone (99.5%, Vetec, Brazil) and CO_2_ (99.9% in a liquid phase, White Martins S.A., Brazil) were used to micronize luteolin. Diazepam (Fagron Pharmaceuticals, Brazil) was used as the positive control. Pentylenetetrazole (PTZ, Sigma-Aldrich, Germany) was used to induce seizures. Commercial kits for RNA extraction (PureLink RNA Mini Kit), RNA and cDNA quantification (Qubit RNA HS Assay Kit and Qubit dsDNA HS Assay Kit), and cDNA synthesis (High-Capacity cDNA Reverse Transcription Kit) were supplied by Thermo Fisher Scientific Inc, United States. PowerUp SYBR Green Master Mix (Invitrogen, United States) was used for quantitative polymerase chain reactions (qPCR). Primers were synthesized by Invitrogen (Brazil).

### 2.2 Micronization

Micronized luteolin was obtained by the gas antisolvent technique (Aguiar et al., 2018; Garbinato et al., 2021; dos Santos et al., 2022; de Oliveira et al., 2023). First, luteolin was briefly mixed in acetone and while being stirred into complete dissolution. The obtained solution was placed in a chamber, and CO_2_ was added to increase the ideal pressure to complete the process. When the pressure was reached, the antisolvent flow was stopped, and the chamber continued to agitate the solution for 10 min at 300 rpm (Aguiar et al., 2018; Garbinato et al., 2021; de Oliveira et al., 2023). After this, the washing stage began with the antisolvent flow rate of 10 mL‧min^−1^, and the pressure was isobaric for 60 min for the complete removal of acetone from the system. The operating conditions were pressure 80 bar and temperature 35 °C. Finally, the obtained material was stored at 4 °C.

### 2.3 Characterization of luteolin and micronized luteolin

Luteolin (raw) and micronized luteolin were characterized by scanning electron microscopy. The particle size was determined using the software Meter Size (version 1.1) (Aguiar et al., 2018; Garbinato et al., 2021; dos Santos et al., 2022; de Oliveira et al., 2023). The melting points of the luteolin and micronized luteolin were verified by a differential scanning calorimeter (Jade-DSC, Perkin Elmer, United States). Samples (5–10 mg) measurements were performed by heating the compounds from 30 °C to 350 °C, at a heating speed of 20 °C‧min^-1^ in an inert atmosphere (N_2_ flow: 20 mL‧min^-1^) (Aguiar et al., 2018; Garbinato et al., 2021; de Oliveira et al., 2023).

### 2.4 Animals

The zebrafish larvae used in this study were obtained from adult zebrafish (*Danio rerio*, AB strain), maintained in our institutional vivarium. Zebrafish were mated, and eggs were obtained following previous protocol (Bertoncello et al., 2018; Decui et al., 2020). Fertilized eggs were collected, freed of debris, and disposed of in sterile cell culture plates containing embryo medium (osmosis water equilibrated with Instant Ocean salts; pH 7.0–7.4). Plates were placed in an incubator at 27 °C, under a 14–10-h light/dark cycle photoperiod (100 lux) until animals reached 5 days post-fertilization (dpf). All experimental practices were approved by the Institutional Ethics Committee for Animal Use (CEUA Unochapecó; Protocol #009/2019) and respected the European Community instructions.

### 2.5 Experimental design

**FIGURE 1:**
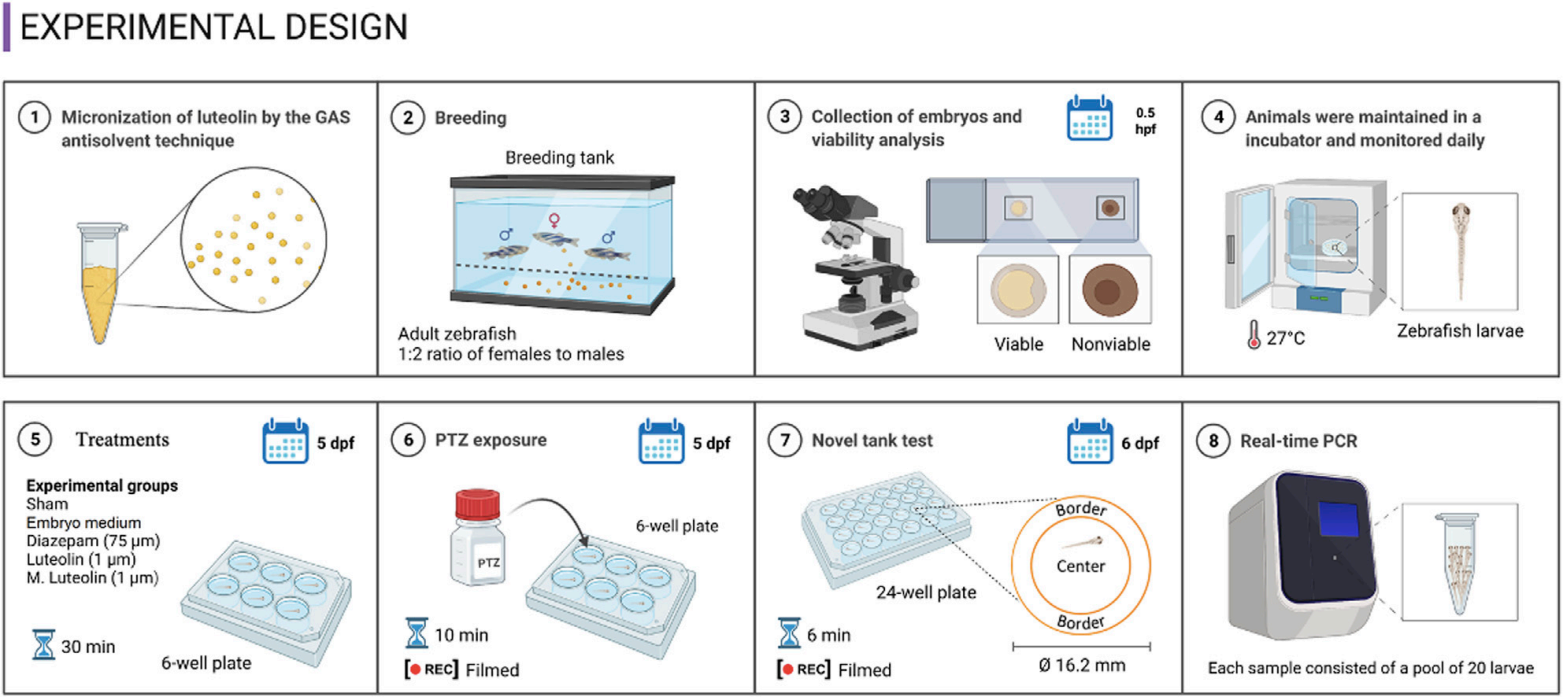
Experimental design.

The protocol, illustrated in Figure 1, consisted of four experimental rounds conducted on different days, totaling 20 animals per experimental group (5 larvae from each group per experimental round) (Bertoncello et al., 2018; Decui et al., 2020). Larvae were randomly allocated into the experimental groups using a computerized random number generator. To improve research reporting, animal housing and experimental procedures followed the ARRIVE guidelines (Percie du Sert et al., 2020). The concentrations of each treatment, the time elapsed between treatment and PTZ exposure, and the PTZ concentration were defined based on previous studies (Bertoncello et al., 2018; Decui et al., 2020).

The experimental groups were: “sham” (animals did not experience any drug treatment and were not exposed to PTZ), “medium + PTZ” (animals were treated with embryo medium and exposed to PTZ), “diazepam + PTZ” (positive control group; animals were treated with diazepam at 75 μM to corroborate the zebrafish response to a clinically used antiseizure drug in the PTZ-induced seizure model), “luteolin + PTZ” (animals were treated with luteolin at 1 μM and exposed to PTZ), and “micronized luteolin + PTZ” (animals were treated with micronized luteolin at 1 μM and exposed to PTZ) (Bertoncello et al., 2018; Decui et al., 2020).

**FIGURE 2:**
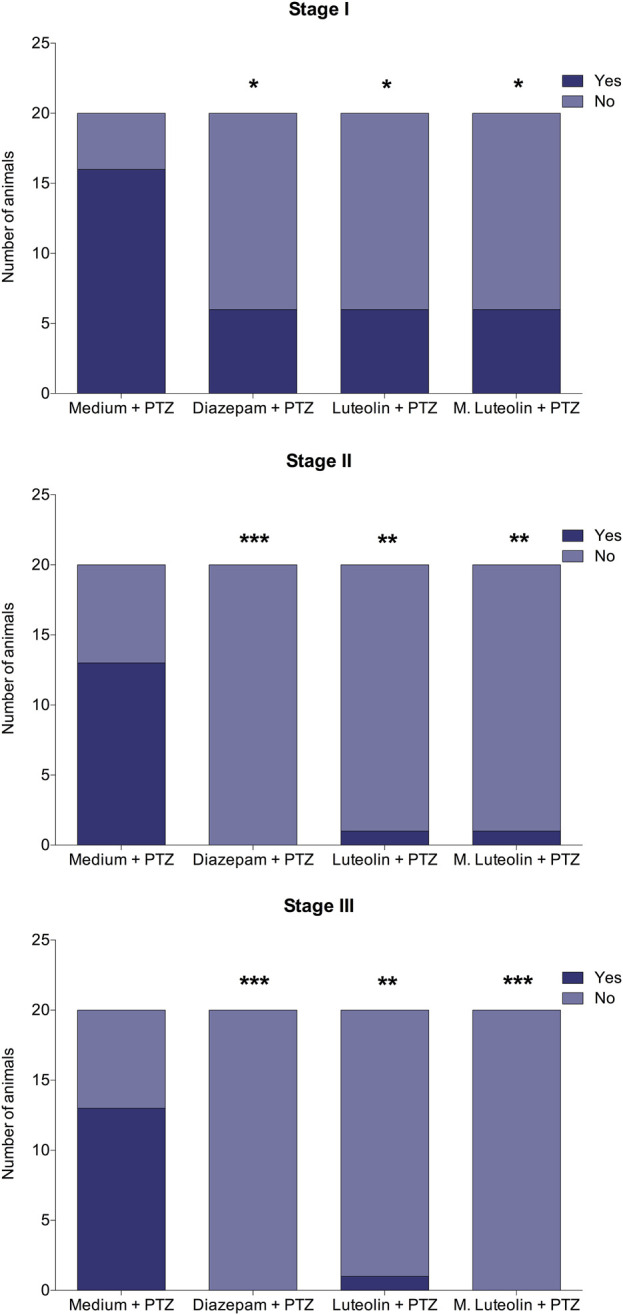
Effects of treatment with embryo medium, diazepam (75 μM), luteolin (1 μM), and micronized luteolin (1 μM) on the occurrence of each seizure stage (I, II, and III) in zebrafish larvae. Data are expressed as the number of animals that reached each seizure stage. The obtained data were analyzed using Fisher’s exact test (n = 20). *p < 0.05, **p < 0.01, and ***p < 0.001 in comparison with “medium + PTZ” group.

**FIGURE 3:**
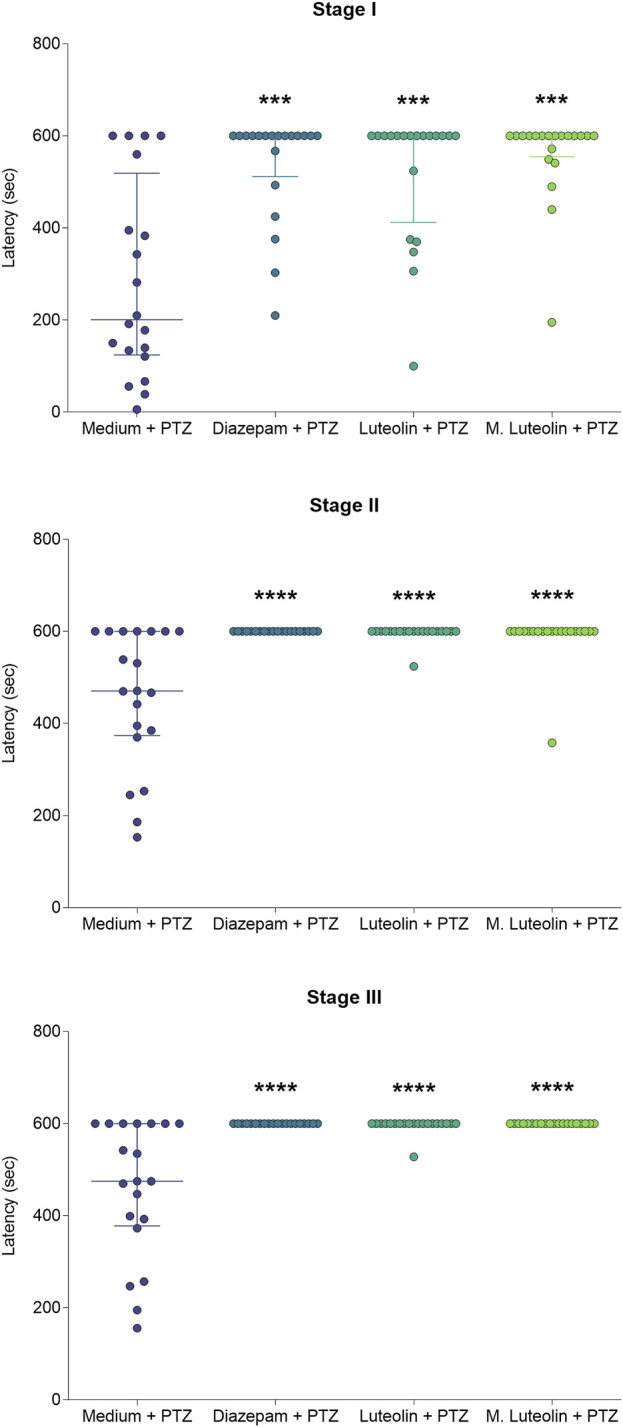
Effects of treatment with embryo medium, diazepam (75 μM), luteolin (1 μM), and micronized luteolin (1 μM) on the latency to reach each seizure stage (I, II, and III) in zebrafish. Data are expressed as median with interquartile range. Data were analyzed by Kruskal–Wallis test (considering treatment as the independent variable), followed by Dunn’s *post hoc* test (n = 20). ***p < 0.001 and ****p < 0.0001 in comparison with “medium + PTZ” group.

**FIGURE 4:**
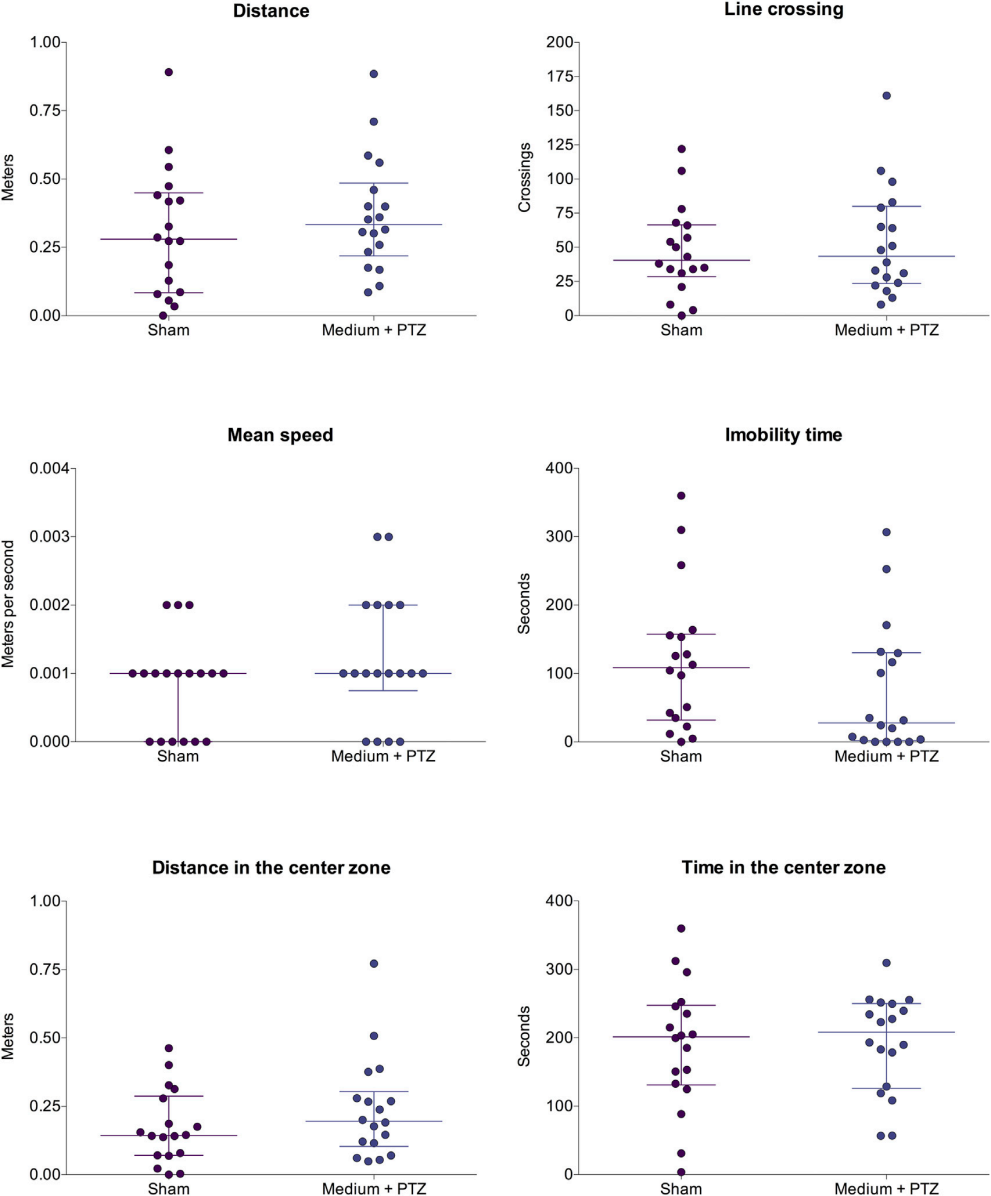
Comparison of larvae’s motor function from “sham” and “medium + PTZ” groups. The “sham” group corresponds to animals not exposed to PTZ or drugs. The “medium + PTZ” group corresponds to animals treated with embryo medium and exposed to PTZ. Data are expressed as median with interquartile range. The obtained data were analyzed by Welch’s t-test (n = 18).

**FIGURE 5:**
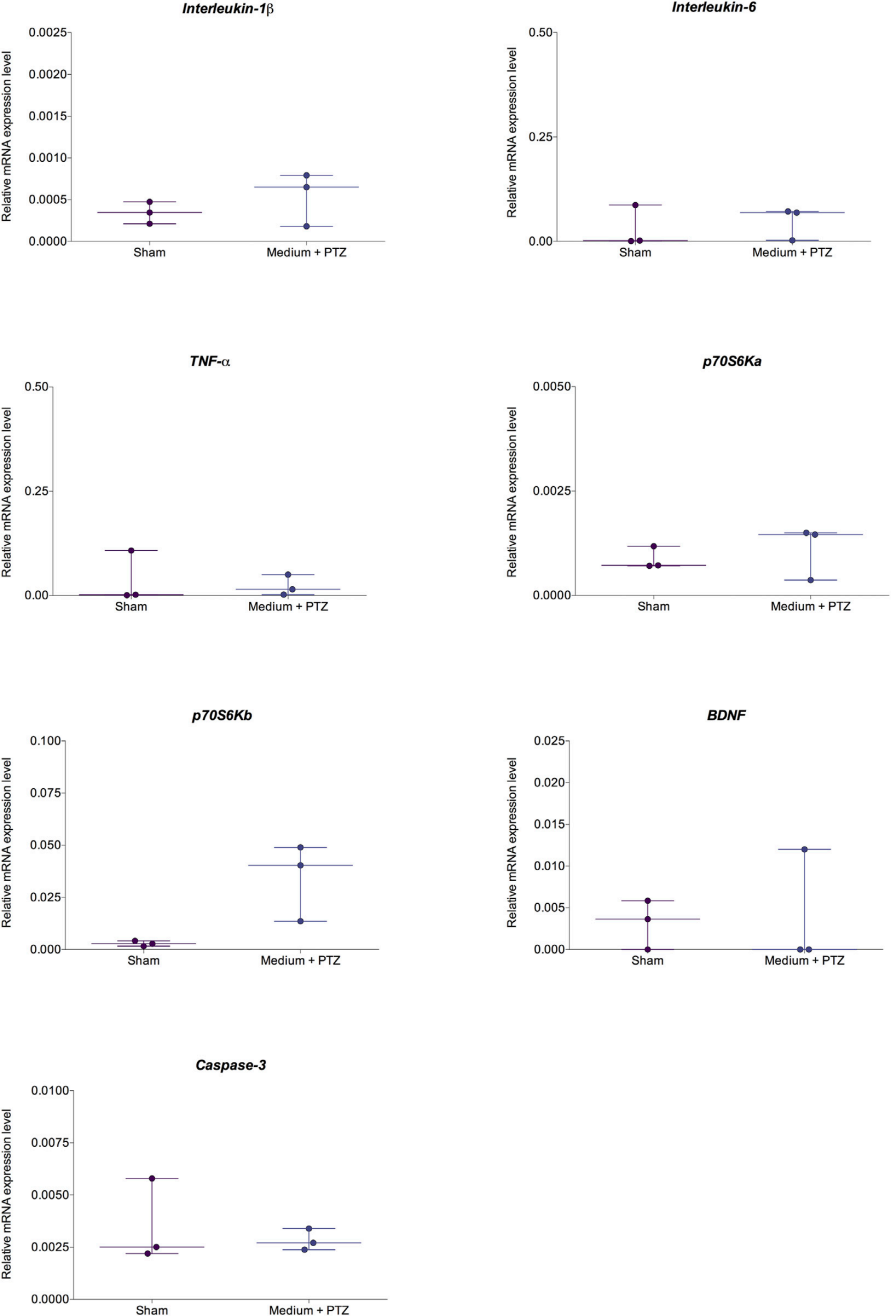
Comparison of relative expression of IL-1β, IL-6, TNF-α, p70S6Ka, p70S6Kb, BDNF, and caspase-3 in zebrafish larvae from “sham” and “medium + PTZ” groups. The “sham” group corresponds to animals not exposed to PTZ or drugs. The “medium + PTZ” group corresponds to animals treated with embryo medium and exposed to PTZ. Data are expressed as median with interquartile range. The obtained data were analyzed by Welch’s t-test (n = 3).

Considering the occurrence of each seizure stage and the latency to reach each seizure stage (Figures 2, 3), the exposed data corresponds to the groups “medium + PTZ”, “diazepam + PTZ”, “luteolin + PTZ”, and “micronized luteolin + PTZ” since the “sham” group did not experience seizures. Considering the animals’ motor function and genetic markers (Figures 4, 5), the exposed data corresponds to “sham” and “medium + PTZ” groups. We intended to verify the neuroprotective potential of luteolin and micronized luteolin through these markers. However, as the seizures did not alter the motor function of the animals as well as the genetic parameters, it would not make sense to investigate the effects of luteolin and micronized luteolin on these biomarkers.

### 2.6 Drug treatments

Each zebrafish larva was exposed to its respective treatment for 30 min prior to PTZ exposure (Bertoncello et al., 2018; Decui et al., 2020). A total of 20 larvae per group were individually placed in 6-well plates (1 larva per well, in 5,000 μL of the corresponding solution) containing embryo medium, diazepam (75 μM), luteolin (1 μM), or micronized luteolin (1 μM) (Bertoncello et al., 2018).

### 2.7 PTZ-induced seizure

Immediately after the treatment, each zebrafish larvae were individually exposed to PTZ solution (3 mM) in 6-well plates (1 larva/well with 5,000 μL of solution) for 10 min. Under these conditions, animals consistently exhibit seizure-like behavior and respond to the positive control drug (Bertoncello et al., 2018; Decui et al., 2020). The animals’ exposure to PTZ was recorded for further analysis. Each video was analyzed by trained and experienced observers blindly. The occurrence of each seizure stage and the latency for the first signal of each seizure stage were evaluated (Bertoncello et al., 2018; Decui et al., 2020). Three convulsive stages were considered: stage I - a dramatic increase in swimming activity; stage II - swimming behavior in whirlpools, and stage III–seizures similar to the clonus state, followed by loss of posture when the animal falls to the side and remains immobile for 1–3 s (Baraban et al., 2005; Bertoncello et al., 2018). After exposure to PTZ, the animals were placed in housing plates (containing embryo medium) according to their respective experimental group. Zebrafish larvae remained in housing plates for 24 h until being tested in the novel tank. The experimental number in the PTZ-induced seizure protocol is 20 (n = 20).

### 2.8 Novel tank test

The animals’ motor function 24 h after PTZ exposure was analyzed by the larvae' novel tank paradigm, adapted from previous studies (Bertoncello et al., 2018; Pedroso et al., 2022). Zebrafish larvae were individually tested in a 24 wells-plate (1 larva/well with 120 μL of embryo medium). The animal locomotion was recorded for 6 min and further analyzed using the ANY-Maze^®^ software (Stoelting Co., Wood Dale, IL, United States).

The analyzed parameters were: total distance traveled, number of line crossings (transitions between the different zones of the plate), mean speed, immobility time, distance traveled in the center zone, and time spent in the center zone. Firstly, we compared larvae that did not experience any drug or PTZ (sham group) with larvae treated with embryo medium and submitted to PTZ-induced seizures (medium + PTZ group). As the seizures did not alter the motor function of the animals, the effects of luteolin and micronized luteolin on biomarkers of motor function were not investigated.

To assess whether luteolin or micronized luteolin could prevent seizure-induced motor changes, we initially compared the profiles of the “Sham” group and the “Medium + PTZ” group. Since no significant differences were observed between these groups, further analyses to investigate the potential effects of luteolin or micronized luteolin on motor function were not performed.

Records presenting interferences (like shadows) that affected the quality of analyses by the ANY-Maze^®^ recording software were discarded. This procedure resulted in two animals per group being removed from the novel tank analyses. The experimental number in the novel tank test is 18 (n = 18).

### 2.9 Real-time PCR

To investigate if the seizure occurrence had any effects on genetic markers of the inflammatory response (*IL-1*β, *IL-6*, and *TNF-*α), mTOR signaling pathway (*p70S6Ka* and *p70S6Kb*), neurogenesis (*BDNF*), and apoptosis (*caspase-3*), we performed quantitative polymerase chain reactions (qPCR) analyses. Firstly, we compared larvae that did not experience any drug or PTZ (sham group) with larvae treated with embryo medium and submitted to PTZ-induced seizures (medium + PTZ group). As the seizures did not alter the analysed transcript levels, the effects of luteolin and micronized luteolin on these biomarkers were not investigated.

For molecular analyses, a second set of 60 animals per experimental group was submitted to the protocol. Zebrafish larvae were cryoanesthetized and euthanized by decapitation after being submitted to the protocol (Gawel et al., 2024). Molecular experiments were performed using n = 3 (Ghaddar et al., 2020; Mazzolini et al., 2020; Pedroso et al., 2022). Each sample consisted of a pool of 20 whole zebrafish larvae (Gawel et al., 2024).

Total RNA was isolated from samples using the PureLink RNA Mini Kit following the manufacturer’s recommendations. The total RNA was quantified by using the Qubit RNA HS Assay Kit. The cDNA was synthesized using the High-Capacity cDNA Reverse Transcription Kit. An average of 0.5 μg of extracted RNA in a reaction with a final volume of 20 μL was used to synthesize cDNA. cDNA quantification was performed by using the Qubit dsDNA HS Assay Kit, and the samples were subsequently diluted to a final concentration of 5 ng‧μL^-1^.

Following the manufacturer’s recommendations, qPCR was performed by using the PowerUp SYBR Green Master Mix. *β-actin* was used as an internal control to normalize the expression of genes of interest (Almeida et al., 2021; Garbinato et al., 2021). Based on our data, the expression of *β-actin* was not altered by treatment, validating its use as an appropriate housekeeping gene for normalization in this study (Tang et al., 2007). All primer sequences are pointed out in Table 1 (Tang et al., 2007; Mei et al., 2008; Frank et al., 2017; van der Vaart et al., 2017; Kirsten et al., 2018; Carty et al., 2019).

**TABLE 1:** Quantitative RT-PCR primers sequences.

Proteins	Primer sequence (5′–3′)
*ß-actin* ^a^	F – CGAGCTGTCTTCCCATCCA R – TCACCAACGTAGCTGCTTTCTG
*IL-1 ß* ^b^	F – GAACAGAATGAAGCACATCAAACC R – ACGGCACTGAATCCACCAC
*IL- 6* ^c^	F – GCGTCCTGACGTGGTATAAAG R – GTCGTTTGGTGCTGTGTTTG
*TNF-*α^c^	F – GACCACAGCACTTCTACCG R – ACATTTTCCTCACTTTCGTTCAC
*p70S6Ka* ^d^	F – ACAGCCCTGATGACACGAAG R – TTCTTGGGCTTCCCAGAACC
*p70S6Kb* ^d^	F – TGACTGATTTCGGGCTGTGT R – CGATTGTGTCCGCTCCTCAT
*BDNF* ^e^	F – GACTCGAAGGACGTTGACCTGTA R – CGGCTCCAAAGGCACTTG
*Caspase*-*3* ^f^	F – TAGTGTGTGTGTTGCTCAGTC R – CTCGACAAGCCTGAATAAAG

^a^ Tang et al., 2007; ^b^ van der Vaart et al., 2017; ^c^ Kirsten et al., 2018; ^d^ Frank et al., 2017; ^e^ Carty et al., 2019; ^f^ Mei et al., 2008.

Each reaction contained 10 ng of cDNA and 0.5 mM of each primer in a final volume of 10 μL. Each sample was analyzed in triplicate. The PCR cycles had the following conditions: 50 °C for 2 min, 95 °C for 10 min, 40 cycles at 95 °C for 15 s, 60 °C for 1 min. The dissociation occurred at 95 °C (1.6 °C‧s^-1^) for 15 s, then 60 °C (1.6 °C‧s^-1^) for 1 min, and, finally, 95 °C (1.6 °C‧s^-1^) for 15 s. The equipment used to perform qPCRs was QuantStudio 3 (Thermo Fisher Scientific). Relative gene expression levels were determined by applying the RQ = 2 ^^ΔΔCt^ method (Livak and Schmittgen, 2001).

To assess whether luteolin or micronized luteolin could prevent seizure-induced gene expression changes, we initially compared the profiles of the “Sham” group and the “Medium + PTZ” group. Since no significant differences were observed between these groups, further analyses to investigate the potential modulatory effects of luteolin or micronized luteolin on gene expression were not performed.

### 2.10 Statistical analysis

First, the normality of the data was analyzed by the Shapiro-Wilk test. To investigate the influence of the treatments (medium, diazepam, luteolin, micronized luteolin; independent variable) on the occurrence of each seizure stage, Fisher’s exact test (two-tailed) was used to carry out pairwise comparisons of all treatments in PAST v.4.03 (Hammer et al., 2001). Pairwise *p*-values were adjusted for multiple comparisons using Bonferroni correction with α = 0.05 (Rice, 1989). The Kruskal–Wallis test, followed by Dunn’s *post hoc* test, was implemented to investigate the influence of the treatments (medium, diazepam, luteolin, micronized luteolin; independent variable) on the latency to reach each seizure stage (dependent variable). To investigate if the seizure occurrence had any effects on motor function and molecular parameters, we performed “sham” and “medium + PTZ” comparisons using Welch’s t-test. Results were considered significant at a *p* < 0.05 level. GraphPad Prism was used to produce graphs. Seizure occurrence data were expressed as the number of animals that reached each seizure stage. Latency, behavioral, and molecular data were expressed as median with interquartile range.

## 3 Results

### 3.1 Profile of luteolin and its micronized form

The scanning electron microscopy analyses (Supplementary Figure S1) showed luteolin presenting irregular particle size and significant agglomeration (Supplementary Figure S1a). Micronized luteolin presented a homogeneous structure (Supplementary Figure S1b). Luteolin has an average particle size of 22.75 µm, and micronized luteolin showed an average size of 2.31 µm.

At the differential scanning calorimeter (Supplementary Figure S2), luteolin showed an endothermic peak at 285.33 °C with ΔH = 38.76 J∙g^-1^, which is characteristic of its melting point. A change in the melting point can be seen in micronized luteolin with the appearance of two endothermic peaks, the first at 249.66 °C with ΔH = 3.23 J∙g^-1^ and the second peak at 321.94 °C with ΔH = 30.67 J∙g^-1^, which indicates a change in the crystalline structure, represented by the change in the melting point of the compound.

### 3.2 Effects of luteolin and micronized luteolin on seizure occurrence and development

The treatments diazepam, luteolin, and micronized luteolin decreased the seizure occurrence (Figure 2). Stage I occurrence was reduced by all treatments compared to medium (negative control) (Bonferroni corrected significance level < 0.05). The occurrence of seizure stage II was reduced by diazepam, luteolin, and micronized luteolin treatments (Bonferroni corrected significance level < 0.001, 0.01, and 0.01, respectively). Finally, the occurrence of the seizure stage III was reduced by diazepam, luteolin, and micronized luteolin treatments (Bonferroni corrected significance level < 0.001, 0.01, and 0.001, respectively).

The treatments diazepam, luteolin, and micronized luteolin slowed the seizure development compared to embryo medium (Figure 3). Diazepam, luteolin, and micronized luteolin treatments increased the latency to stage I (Kruskal–Wallis statistic = 23.74; *p* < 0.0001), II (Kruskal–Wallis statistic = 37.06; *p* < 0.0001), and III (Kruskal–Wallis statistic = 41.80; *p* < 0.0001).

### 3.3 Effects on locomotor function

There were no differences among the “sham” and “medium + PTZ” groups (Figure 4) for total traveled distance (Welch corrected *t* = 0.854; df = 33.55; *p* = 0.143), line crossing (Welch corrected *t* = 0.560; df = 32.79; *p* = 0.197), mean speed (Welch corrected *t* = 1.400; df = 31.53; *p* = 0.225), immobility time (Welch corrected *t* = 1.350; df = 33.63; *p* = 0.063), distance traveled in center zone (Welch corrected *t* = 1.213; df = 31.10; *p* = 0.264), and time spent in center zone (Welch corrected *t* = 0.132; df = 32.05; *p* = 0.134).

### 3.4 Effects on molecular markers

There were no differences between the “sham” and “medium + PTZ” groups for molecular results (Figure 5). Our results show that there were no changes in the *IL-1*β (Welch corrected *t* = 0.990; df = 2.654; *p* = 0.403), *IL-6* (Welch corrected *t* = 0.487; df = 3.797; *p* = 0.652), and *TNF- α* (Welch corrected *t* = 0.375; df = 2.631; *p* = 0.735) transcript levels. Also, there were no changes in the *p70S6Ka* (Welch corrected *t* = 0.594; df = 2.677; *p* = 0.598) and *p70S6Kb* (Welch corrected *t* = 2.943; df = 2.019; *p* = 0.097) transcript levels. Finally, there were no changes (Figure 5) in the *BDNF* (Welch corrected *t* = 0.191; df = 2.705; *p =* 0.861) and *caspase-3* (Welch corrected *t* = 0.563; df = 2.266; *p* = 0.623) transcript levels in zebrafish larvae 24 h after PTZ-induced seizures.

## 4 Discussion

Here, we aimed to investigate the antiseizure and neuroprotective potential of luteolin and micronized luteolin in developing zebrafish. Both luteolin and micronized luteolin reduced the occurrence of each seizure stage and slowed the seizure development. There were no residual effects on larvae’ motor function 24 h after the PTZ-induced seizures. Finally, the transcript levels of genetic markers of the inflammatory response (*IL-1*β, *IL-6*, and *TNF-*α), mTOR signaling (*p70S6Ka* and *p70S6Kb*), neurogenesis (*BDNF*), and apoptosis (*caspase-3*) were not altered 24 h after seizure occurrence. These findings represent the first report on luteolin’s anti-seizure properties in larval zebrafish and contrast with previously published work on adult zebrafish. Garbinato et al. (2021) showed that neither luteolin at 0.5 mg‧ kg^-1^ nor micronized luteolin at 0.5 mg‧kg^-1^ reduced PTZ-induced seizures in adult zebrafish. This discrepancy may reflect developmental differences in blood-brain barrier permeability, pharmacokinetics, and receptor expression and highlights the importance of the developmental stage when evaluating drug efficacy. In zebrafish larvae, particularly at 5 dpf, the blood-brain barrier is still maturing (Fleming et al., 2013; Quiñonez-Silvero et al., 2020), possibly allowing more effective brain penetration of luteolin.

In rats, luteolin exerted a dose-dependent antiseizure effect. Luteolin reduced PTZ-induced seizure severity, delayed seizure development, and shortened seizure duration. Also, it promoted neuroprotective effects, preventing hippocampal neuronal damage and partially restoring behavioral function and learning, and memory abilities in the PTZ-induced epilepsy model. However, luteolin treatment did not prevent the seizure occurrence (Cheng et al., 2024). Noteworthy, luteolin attenuates PTZ-induced cognitive deficits in rats by reducing oxidative stress and activating the PKA/CREB/BDNF signaling axis—a pathway critically involved in learning, memory, and neuronal survival (Zhen et al., 2016). Although we did not detect changes in BDNF transcript levels, it is possible that earlier or more dynamic transcriptional events occurred outside our sampling window, or that post-transcriptional regulation contributed to neuroprotection.

Critical transcriptional changes related to the seizure response might occur at earlier time points (1–6 h) after the episode (Fleming et al., 2013; Quiñonez-Silvero et al., 2020). Equally, cell condition, neuroinflammatory or neuroplasticity-related (mTOR) molecular changes may peak later, beyond 24 h post-seizure (Talos et al., 2012; Hodges and Lugo, 2020). Previous studies have implicated these pathways in seizure propagation, particularly in drug-resistant epilepsy (Hodges and Lugo, 2020; Ravizza et al., 2024).

As part of a class of flavonoids with pleiotropic activity (including curcumin, quercetin, and resveratrol), luteolin has been implicated in regulating multiple pathways simultaneously, such as inhibiting inflammation and downregulating mTOR. These multimodal actions could underlie its broad efficacy observed in this and other models. Although luteolin is well known for its anti-inflammatory properties and ability to modulate mTOR signaling (Zhou et al., 2021; Cheng et al., 2024; Goyal et al., 2023), we did not observe significant alterations in the expression of inflammatory cytokines (IL-1β, IL-6, TNF-α) or mTOR pathway genes (p70S6Ka, p70S6Kb) 24 h after seizure induction by exposure to PTZ at 3 mM. This suggests that, at 5 dpf, PTZ (3 mM)-induced seizures may not be sufficient to activate prolonged inflammatory or mTOR-related transcriptional changes, or that such changes occur earlier or later than the assessed time point. Furthermore, the neurodevelopmental stage of zebrafish larvae, characterized by an immature immune system, may influence the regulation of these pathways (Fleming et al., 2013; Quiñonez-Silvero et al., 2020). It is possible that the timing of molecular analysis failed to capture the transient alterations in these pathways. Future investigations should explore earlier or later time points and different concentrations of PTZ to determine whether luteolin’s effects involve short-term modulation of neuroinflammatory and cell signaling processes.

Micronization technology has been applied to improve the bioavailability of flavonoids (Aguiar et al., 2018; Kurniawansyah et al., 2015; de Oliveira et al., 2023). Despite the encouraging findings regarding the antiseizure effects of luteolin in zebrafish larvae, we failed to observe a clear advantage of micronized luteolin over its raw form. Polyphenols’ pharmacological potential could be enhanced by increasing their bioavailability through the micronization process (de Oliveira et al., 2023). In adult zebrafish and zebrafish larvae at 7 dpf micronized curcumin and resveratrol showed an increased effect in seizure control compared to raw compounds (Bertoncello et al., 2018; Decui et al., 2020; Almeida et al., 2021). Our results demonstrate that the micronization process reduced the luteolin particle size, and smaller particles may be a factor that can contribute to the increase in bioavailability. However, under the present experimental conditions, no clear differences were observed between raw and micronized luteolin in modulating seizure-related outcomes in zebrafish larvae at 5 dpf. The absence of significant effects may be related to the concentration used (1 μM). Although previous studies in zebrafish larvae reported promising antiseizure effects of polyphenols at this concentration (Bertoncello et al., 2018; Decui et al., 2020), the same response was not reproduced here. Therefore, future studies should investigate a broader concentration–response range to determine whether luteolin’s efficacy is concentration-dependent.

At least in part, the absence of effects can be attributable to the immature blood brain barrier (BBB) in 5 dpf larvae (Fleming et al., 2013; Quiñonez-Silvero et al., 2020), which could reduce the relevance of solubility-related enhancements. As such, while larval zebrafish with an immature BBB offer a valuable early-stage screening platform, the investigation of polyphenol efficacy in seizure models using adult zebrafish deserves attention. A previous study showed that neither luteolin nor micronized luteolin exhibited antiseizure effects in adult zebrafish (Garbinato et al., 2021), highlighting potential developmental differences in pharmacokinetics and treatment response.

One limitation of the present study is the absence of pharmacokinetic data on luteolin and micronized luteolin. The preparation of nanoparticles improved luteolin’s solubility in water and increased its bioavailability and antioxidant effects (Wang et al., 2019). Furthermore, a recent study demonstrated that luteolin–phospholipid complexes significantly improved luteolin’s oral bioavailability and increased its renoprotection properties (Mao et al., 2025). These results supported the relevance of bioavailability-enhancing strategies for optimizing luteolin’s therapeutic potential. While their antiseizure effects indicate promising therapeutic potential, determining their plasma concentrations and brain penetration is crucial for assessing their antiseizure viability. Further studies incorporating bioavailability and tissue distribution analyses will strengthen our understanding of their pharmacological properties.

Overall, our findings demonstrate for the first time that both luteolin and micronized luteolin effectively reduce seizure occurrence and severity in zebrafish larvae. The results observed here, integrated with prior literature, support luteolin’s potential as a multifunctional neuroprotective agent and provide a foundation for future studies aimed at elucidating its therapeutic efficacy in epilepsy during early developmental stages.

## Data Availability

All data are available in the Open Science Framework (https://osf.io/4gax5). Further inquiries can be directed to the corresponding author.
