# Supplementary figures and images for: Zebrafish-based assessment of luteolin’s potential in modulating seizure responses
Source: Front Pharmacol. 2025 Aug 29;16:1656301. doi: 10.3389/fphar.2025.1656301 (PMC12426234; doi:10.3389/fphar.2025.1656301)

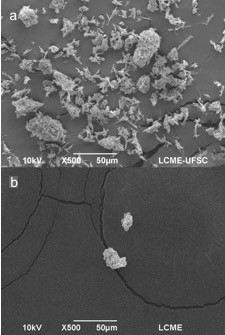

Supplement: Supplementary file 1 [file Image1.tiff]

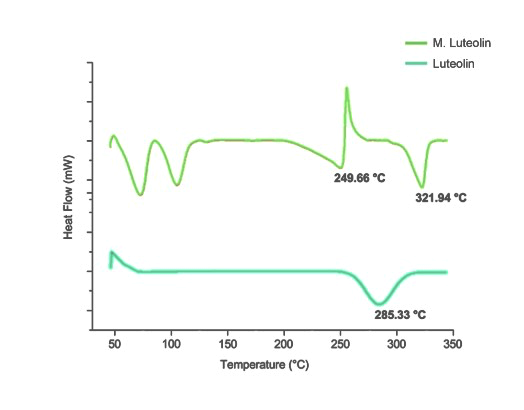

Supplement: Supplementary file 2 [file Image2.tiff]
